# Lactate and Lactate-to-Pyruvate Ratio in Critically Ill COVID-19 Patients: A Pilot Study

**DOI:** 10.3390/jpm12020171

**Published:** 2022-01-27

**Authors:** Alice G. Vassiliou, Stamatios Tsipilis, Chrysi Keskinidou, Charikleia S. Vrettou, Edison Jahaj, Parisis Gallos, Christina Routsi, Stylianos E. Orfanos, Anastasia Kotanidou, Ioanna Dimopoulou

**Affiliations:** 1 First Department of Critical Care Medicine & Pulmonary Services, School of Medicine, National & Kapodistrian University of Athens, Evangelismos Hospital, 106 76 Athens, Greece; alvass75@gmail.com (A.G.V.); stamostsipil@gmail.com (S.T.); chrysakes29@gmail.com (C.K.); vrettou@hotmail.com (C.S.V.); edison.jahaj@gmail.com (E.J.); chroutsi@med.uoa.gr (C.R.); sorfanos@med.uoa.gr (S.E.O.); akotanid@med.uoa.gr (A.K.); 2 Computational Biomedicine Laboratory, Department of Digital Systems, University of Piraeus, 185 34 Piraeus, Greece; parisgallos@unipi.gr

**Keywords:** COVID-19, critically ill, lactate, pyruvate, LP ratio, mortality, metabolic pattern

## Abstract

A limited number of coronavirus disease-19 (COVID-19) cases may require treatment in an intensive care unit (ICU). Arterial blood lactate levels are routinely measured in the ICU to estimate disease severity, predict poor outcomes, and monitor therapeutic handlings. A number of studies have suggested that, simultaneously with lactate, pyruvate should also be measured, providing augmented prognostic ability, and a better understanding of the underlying metabolic alterations in ICU patients. Hence, the aim of the present study was to elucidate the relationship between lactate levels and the lactate-to-pyruvate (LP) ratio with the clinical outcome in mechanically ventilated COVID-19 patients. Lactate and pyruvate were serially measured during the first 24 h of ICU stay. A group of ICU non-COVID-19 patients was used as a comparison group. The majority of COVID-19 patients (82.5%) had normal lactate levels and a normal LP ratio on ICU admission (normal metabolic pattern). A small, yet significant, percentage of patients had either elevated lactate levels or a high LP ratio (abnormal metabolic pattern); these patients exhibited a significantly higher risk of ICU mortality compared to the patients with a normal metabolic pattern (72.7% vs. 34.6%, *p* = 0.04). In our critically ill COVID-19 patients, elevated lactate levels or high LP ratios on admission to the ICU could be associated with poor clinical outcome.

## 1. Introduction

Coronavirus disease-19 (COVID-19) usually manifests with mild symptoms and signs. However, a number of cases (5–10%) may deteriorate, resulting in pneumonia, acute respiratory distress syndrome (ARDS), and multi-organ failure, requiring treatment in an intensive care unit (ICU) for invasive monitoring and mechanical ventilation [1].

Increased blood lactate is a common finding in critically ill patients [2]. Traditionally, this is considered a surrogate for oxygen delivery to tissues; this metabolite, however, is the result of a complex interplay between tissue metabolic demands, oxygen supply, and blood clearance. In non-hypoxic states, increased blood lactate concentrations may be due to other factors, such as increased aerobic glycolysis [3,4], or insufficient pyruvate dehydrogenase (PDH) activity [5]. Arterial blood lactate levels are routinely measured in the ICU to estimate disease severity, predict morbidity and mortality, indicate specific treatments, and monitor the adequacy and timing of interventions [2]. It has been proposed that evaluation of the time course of blood lactate concentrations may yield more information than a single blood lactate measurement [6].

A number of studies in critically ill patients have suggested that, simultaneously with lactate, its precursor pyruvate should also be measured [7,8,9,10]. The combined measurement of the two metabolites might augment prognostic ability, and provide a better understanding of the underlying metabolic alterations in ICU patients. The early work of Weil and Afifi showed that evaluating the lactate-to-pyruvate (LP) ratio added no value to lactate levels in predicting survival from circulatory shock [10]. On the contrary, more recent studies revealed that the combination of elevated lactate and LP ratio is associated with increased mortality in an unselected population of critically ill patients [9], and in patients with septic shock [7,8]. The LP ratio has also been studied in a limited number of clinical settings to help differentiate between hypoxic and non-hypoxic hyperlactatemia [8]. In non-hypoxic conditions, hyperlactatemia occurs with a normal LP ratio, whereas a simultaneous elevation of lactate and the LP ratio indicates tissue hypoxia [11]. The potential role and utility of the LP ratio in the setting of ICU COVID-19 patients has not been investigated.

In light of the above, we designed a prospective observational study in order to elucidate the relationship between lactate levels and the LP ratio with ICU mortality in mechanically ventilated COVID-19 patients. To this end, lactate and pyruvate were serially measured during the first 24 h of ICU stay. A group of ICU non-COVID-19 patients was additionally studied. We also analyzed the subgroup of COVID-19 patients presenting with elevated lactate in an attempt to assess the contribution of hypoxic and non-hypoxic causes of hyperlactatemia. In order to evaluate the clinical utility of the LP ratio as a biomarker, we also measured other potentially relevant serum biomarkers in this setting, such as interleukin (IL)-6, IL-8, IL-10 and tumour necrosis factor (TNF)-α.

## 2. Materials and Methods

The study was approved by the ‘Evangelismos’ Hospital Research Ethics Committee (73/26-2-2020) and all procedures carried out on patients were in compliance with the Helsinki Declaration. Informed written consent was obtained from all patients’ next-of-kin. 

The present study included 63 consecutive, mechanically ventilated adult COVID-19 critically ill patients, hospitalized in the ICU of the ‘Evangelismos’ General Hospital from 30 September 2020 to 18 May 2021. Severe acute respiratory syndrome coronavirus 2 (SARS-CoV-2) infection was verified by real-time reverse transcription PCR (RT-PCR) in nasopharyngeal swabs. An additional group of 17 mechanically ventilated adult general ICU patients was included for comparison purposes. Patients who did not require mechanical ventilation during ICU stay were not included in the study. No other exclusion criteria were applied. The numerical imbalance between COVID-19 and non-COVID-19 ICU patients was due to the fact that by mid-November all our ICUs were occupied by COVID-19 patients. Following study enrolment, demographic characteristics, comorbidities, and laboratory findings were recorded. Acute Physiology and Chronic Health Evaluation (APACHE II) and Sequential Organ Failure Assessment (SOFA) scores were calculated on ICU admission (within 24 h). Sepsis and septic shock were defined according to Sepsis-3 guidelines [12]. Outcome was defined as ICU mortality.

Blood lactate was measured 4 times daily within the first 24 h from ICU admission. A blood gas analyzer (Radiometer ABL 700 series, Radiometer Medical APS, Copenhagen, Denmark) was used for lactate measurements.

Blood pyruvate was measured 4 times daily within the first 24 h from ICU admission, concurrently with lactate measurements. Pyruvate was measured spectrophotometrically using the Pyruvic Acid Assay kit, according to the manufacturer’s protocol (Megazyme, Wicklow, Ireland). More specifically, the blood samples were deproteinized by adding an equal volume of ice-cold 1 M perchloric acid with mixing. The samples were centrifuged, and the recovered supernatant was used in the assay.

The lactate-to-pyruvate (LP) ratio was calculated thereafter for each patient, and at every time-point studied. Based on lactate levels and LP ratio, patients were assigned to either of 2 metabolic pattern groups; normal (lactate values ≤ 2 mmol/L and LP ratio ≤ 10) or abnormal (high lactate or high LP ratio) [11].

ICU admission (within 24 h) serum concentrations of interleukin (IL)-6, IL-8, IL-10, and tumour necrosis factor (TNF)-α were measured by enzyme-linked immunosorbent assays (ELISA) (Invitrogen, Thermo Fisher Scientific, Waltham, MA USA), in the 80 critically ill patients enrolled in the study. 

Data are presented as mean ± standard deviation (SD) for normally distributed variables or as median with inter-quartile range (Q1–Q3) for skewed data. The two-group comparisons were performed by the t-test or the non-parametric Mann-Whitney test, as appropriate. Associations between qualitative variables were examined by the chi-square test. The Hedges’ g effect size was calculated for normally distributed data (mean difference divided by pooled weighted standard deviation), Cohen’s d for skewed data (by dividing the z-value of the Mann-Whitney test by the square root of the number of subjects), and phi for qualitative variables [13]. Correlations were performed by Spearman’s correlation coefficient. Mixed-effect models were used to describe the time course of lactate and the LP ratio. Models included the two groups, i.e., COVID-19 infection or not, time and their interaction. A univariate logistic regression model was used to evaluate the association of the metabolic pattern with ICU mortality risk. Afterwards, a multivariate logistic regression model was performed to evaluate the association of the metabolic pattern with ICU mortality risk in the presence of potential confounders, namely age, lactate levels on ICU admission, and APACHE II score, i.e., those covariates that were significantly different between survivors and non-survivors. The analyses were performed with IBM SPSS statistical package, version 22 (IBM Software Group, Armonk, NY, USA), and GraphPad Prism, version 8.0 (GraphPad Software, San Diego, CA, USA). All p-values are two-sided; *p* < 0.05 was considered significant. Effect size measures the magnitude of the experimental effect. The larger the effect size the stronger the relationship between two variables. According to Cohen, d and phi values equal to 0.1 are considered a ‘small’ effect size, 0.3 represents a ‘medium’ effect size and 0.5 a ‘large’ effect size. For Hedge’s g values, 0.2 is considered a small effect size, 0.5 a medium effect size, and 0.8 a large effect size.

## 3. Results

### 3.1. Study Population

All patients were mechanically ventilated. The COVID-19 patients received dexamethasone (6 mg/day) as part of their treatment. Clinical characteristics and laboratory data in critically ill COVID-19 and non-COVID-19 patients on admission to the ICU are presented in Table 1. Patients’ outcome and demographics are also shown. On ICU admission, critically ill COVID-19 and non-COVID-19 patients did not differ in terms of age, sex, underlying comorbidities, or critical illness severity, as expressed by the APACHE II score (*p* > 0.05). The APACHE II score is a general measure of disease severity based on current physiological measurements, age and previous health conditions. On admission to the ICU, lactate levels were similar in the two groups (*p* = 0.60), but the LP ratio was lower in critically ill COVID-19 patients (*p* = 0.005). COVID-19 patients required less vasopressors (mean dose 0.25 µg/kg/min vs. 0.45 µg/kg/min, *p* = 0.03 and maximum dose 0.25 µg/kg/min vs. 0.45 µg/kg/min, *p* = 0.01). Lactate dehydrogenase (LDH) was higher in COVID-19 patients (497 U/L vs. 254 U/L, *p* = 0.001). Only one non-COVID-19 patient and 2 COVID-19 patients received glucose intravenous infusion (*p* = 0.53). The fluid balance was similar in both groups (1428 (279–4057) vs. 1957 (1050–3093); *p* = 0.92, respectively). As far as cytokines are concerned, there was a significant difference only in IL-6 levels between the two groups. Specifically, non-COVID-19 patients had higher IL-6 levels (36.6 pg/mL vs. 16.1 pg/mL, *p* = 0.01). This might be due to the fact that the COVID-19 patients received dexamethasone. Finally, ICU mortality was similar in the two groups (*p* > 0.05).

### 3.2. Lactate and Lactate-to-Pyruvate Ratio in COVID-19 and Non-COVID-19 Patients

**Figure 1:**
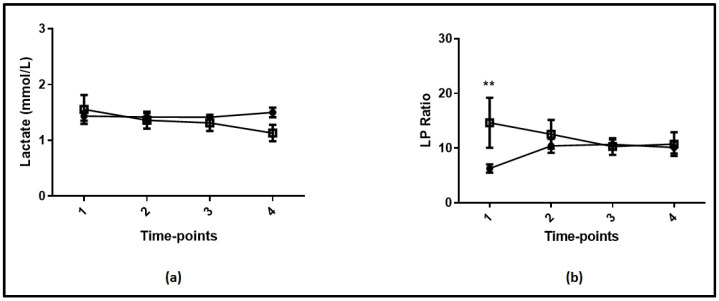
(**a**) Lactate levels at each time-point studied in COVID-19 and non-COVID-19 critically ill patients. (**b**) Lactate-to-pyruvate (LP) ratio at each time-point studied in COVID-19 and non-COVID-19 critically ill patients. Analysis was performed using mixed model fixed effects for lactate levels and the LP ratio over the first 24 h of ICU stay and COVID-19 infection as the grouping factor. Open square, non-COVID-19 critically ill patients; closed circle, COVID-19 critically ill patients. **—*p* < 0.01 between COVID-19 and non-COVID-19 patients.

In Figure 1, the time courses of lactate (A) and the LP ratio (B) are given in the critically ill COVID-19 and non-COVID-19 patients. There were no differences in lactate levels between the two groups at any time-point studied (Figure 1a; *p* > 0.05). The two groups had different LP ratios only on admission to the ICU (Figure 1b and Table 1; *p* = 0.005).

### 3.3. Lactate and Lactate-to-Pyruvate Ratio in COVID-19 Survivors and Non-Survivors

**Figure 2:**
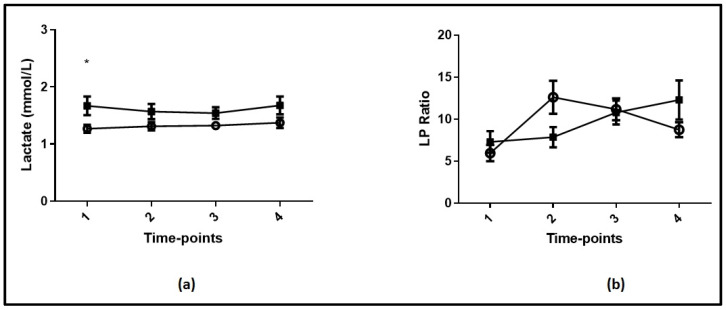
(**a**) Lactate levels at each time-point studied in COVID-19 survivors and non-survivors. (**b**) Lactate-to-pyruvate (LP) ratio at each time-point studied in COVID-19 survivors and non-survivors. Analysis was performed using mixed model fixed effects for lactate levels and the LP ratio over the first 24 h of ICU stay and ICU mortality as the grouping factor. Open circle, COVID-19 survivors; closed square, COVID-19 non-survivors. *—*p* < 0.05 between survivors and non-survivors.

The time course of lactate levels and the LP ratio in COVID-19 survivors and non-survivors during the four time-points are shown in Figure 2. As seen, lactate levels differed between critically ill COVID-19 survivors and non-survivors only on ICU admission (Figure 2a and Table 2; *p* = 0.01). There were no differences in the LP ratio (Figure 2b) between critically ill COVID-19 survivors and non-survivors at any time-point studied (*p* > 0.05). The characteristics of COVID-19 survivors and non-survivors are shown in Table 2. The two sub-groups differed in terms of age (*p* = 0.006), APACHE II score (*p* = 0.002), and ICU admission lactate levels (*p* = 0.01). The ICU admission LP ratio tended to be higher in the non-survivors (*p* = 0.056).

### 3.4. Metabolic Patterns in COVID-19 Patients and ICU Mortality

**Figure 3:**
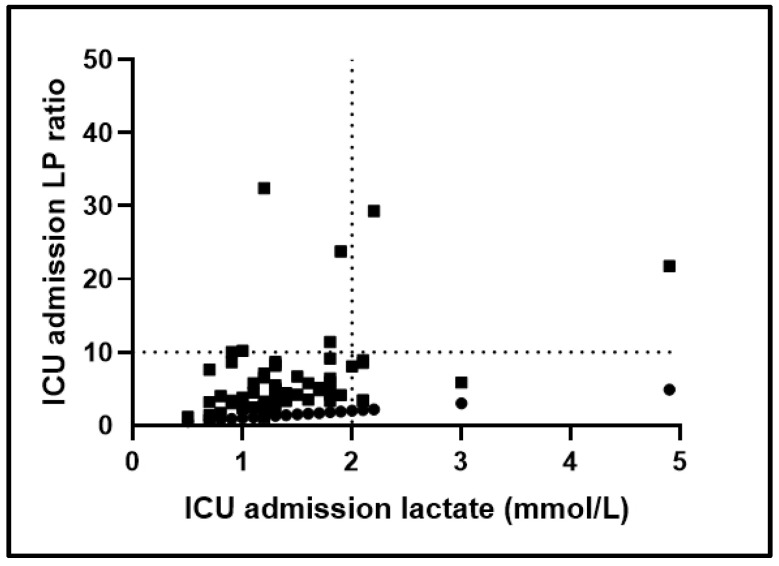
Plot of ICU admission lactate levels and lactate-to-pyruvate (LP) ratio in COVID-19 patients. The LP ratios are given in the *y*-axis and lactate levels on the *x*-axis. Horizontal line, LP ratio = 10; vertical line, lactate = 2 mmol/L.

The plot between ICU admission lactate levels and ICU admission LP ratio is shown in Figure 3. As seen, 52 critically ill COVID-19 patients (82.5%) had normal lactate values (≤2 mmol/L) and a normal LP ratio (≤10) on ICU admission (normal metabolic pattern). The remaining 11 patients (17.5%) had either high lactate levels (>2 mmol/L) or a high LP ratio (>10) on ICU admission (abnormal metabolic pattern). Of these 11 patients, five patients (7.9%) had high lactate levels and a normal LP ratio, two (3.2%) had high lactate levels and a simultaneously elevated LP ratio, while the remaining four (6.4%) patients had a high LP ratio and normal lactate levels. ICU mortality in the normal metabolic pattern group was 34.6% (18/52 patients), as opposed to 72.7% (8/11) in the patients with an abnormal metabolic pattern (*p* = 0.04). Patients with an abnormal metabolic pattern had a significantly higher APACHE II score compared to those with a normal metabolic pattern (16 (14–23) vs. 13 (11–16); *p* = 0.02), and tended to have higher IL-6 levels (47.2 (7.9–155.4) pg/mL vs. 12.7 (8.7–31.5) pg/mL; *p* = 0.05).

Table 3 shows the results of the univariate and multivariate analyses. The univariate analysis indicated that an abnormal metabolic pattern was associated with a higher ICU mortality risk (OR = 6.447, 95% CI = 1.216–34.173; *p* = 0.029). The multivariate model included other possible independent confounders, namely age, lactate levels on ICU admission, and APACHE II score. The multivariate analysis showed that an abnormal metabolic pattern could not be assumed as an independent predictor of ICU mortality, in the presence of age, ICU admission lactate levels, and APACHE II score.

In the critically ill non-COVID-19 control group, nine patients (53%) had a normal metabolic pattern, while the remaining eight (47%) had an abnormal metabolic pattern. ICU mortality in these two subgroups did not differ (33.3% vs. 37.5%; *p* > 0.9). This could be due to the limited number of patients.

## 4. Discussion

In the present investigation, we serially measured lactate and pyruvate during the first 24 h of ICU stay in a group of mechanically ventilated COVID-19 patients. The findings of the current study can be summarized as follows: (i) the majority of COVID-19 patients had normal lactate levels and LP ratios on admission to the ICU; there was, however, a small, yet significant percentage of patients (~20%) who had either elevated lactate levels or a high LP ratio; these patients exhibited a significantly higher ICU mortality risk. (ii) high lactate levels seemed to be related to hypoxic or non-hypoxic causes in COVID-19 patients.

Glycolysis, the major pathway of glucose metabolism, occurs in the cytosol. Under aerobic conditions, mitochondria generate adenosine triphosphate (ATP) for metabolism. During this process, glucose is converted to pyruvate, and through a series of chemical reactions, the stored energy is released via the oxidation of acetyl-coenzyme A. Under anaerobic conditions however, pyruvate is shunted towards the production of lactate by the enzyme lactate dehydrogenase (LDH). 

The LP ratio is a reliable index of cellular oxygenation adequacy [8,9]; clinical experience with this ratio is limited, since the measurement of pyruvate is technically demanding. Once the sample is drawn, pyruvate is converted to lactate by the action of LDH. Hence, processing should be performed immediately, otherwise a lower value in pyruvate can be obtained, increasing the LP ratio. An early study showed no benefit of measuring the LP ratio in addition to lactate levels in patients with circulatory shock, in terms of outcome prediction [10]. Subsequently, a limited number of investigations highlighted the importance of concurrently measuring lactate and pyruvate. In a prospective study on emergency admission patients, those with hyperlactatemia and simultaneously elevated LP ratios (>18:1) had substantially higher in-hospital mortality, compared to patients with isolated hyperlactatemia (37.5% vs. 12.5%) [9]. A later study measured serum lactate and pyruvate in shock patients every 4 h after admission. The study showed an increased LP ratio in the majority of patients with septic or cardiogenic shock. Higher LP ratios were additionally found at shock onset in non-survivors compared to survivors (24 vs. 15). The authors suggested that the LP ratio confirmed that in shock states hyperlactatemia is frequently, but not solely, due to hypoxia, and that an elevated ratio is associated with poor outcome [8]. These findings were corroborated by another study, which demonstrated that patients with septic shock who died within the first 24 h of admission had average LP ratios of 37 compared to those who died later, and survivors (20 and 14, respectively). Furthermore, the same study showed that the LP ratio decreased significantly in survivors during the first 24 h, while remained unchanged in non-survivors [7]. 

Our group recently demonstrated that initial blood lactate served as an independent outcome predictor in critically ill COVID-19 patients. Moreover, the time course of lactate mirrored organ dysfunction, and was associated with poor clinical outcomes [14]. In the present study we measured pyruvate concomitantly with lactate to assess the potential utility of the LP ratio in COVID-19 ICU patients. We also included a group of non-COVID-19 ICU patients who served as the control group. We used a validated spectroscopic assay for pyruvate measurements. Controls required more vasopressors, and had a higher LP ratio, yet similar lactate levels on ICU admission compared to COVID-19 patients. In subsequent time-points, the LP ratio did not differ between the two groups; this might be attributed to the resuscitation handlings performed on patients. It is worth noticing that IL-6 levels were also higher in the controls compared to the COVID-19 patients; however, the latter were receiving dexamethasone as per international guidelines [15]. We subsequently analyzed COVID-19 patients based on their admission lactate levels and LP ratio. Patients were assigned to either of two metabolic pattern groups; normal (lactate values ≤ 2 mmol/L and LP ratio ≤ 10) or abnormal (lactate > 2 mmol/L or LP ratio > 10). Most patients (82.5%) had a normal pattern, while the remaining 17.5% had an abnormal pattern, which was characterized by either high lactate levels or a high LP ratio. The patients with an abnormal pattern exhibited significantly higher mortality rates compared to patients with a normal metabolic pattern (72.7% vs. 34.6%, respectively). Overall, seven patients had high lactate, and of these only in two was the LP ratio elevated. The traditional view is that hyperlactatemia is attributed to anaerobic metabolism due to inadequate perfusion and oxygen delivery to the tissues [3]. Our data suggest that other non-hypoxic processes may also increase lactate levels in ICU COVID-19 patients, supporting the theory that the etiology of hyperlactatemia in severely ill patients is probably multifactorial [3]. These include increased aerobic glycolysis caused by beta-adrenergic stimulation related to vasopressor or inotrope use, inhibition of PDH, or increased protein degradation causing amino acid conversion to pyruvate [8]. In our population, non-COVID-19 ICU patients presented with higher LP ratios; this could be attributed to the higher vasopressor dose administered (Figure 1B and Table 1). Of interest, four COVID-19 patients had an elevated LP ratio with normal lactate levels, suggesting increased rate of pyruvate utilization. The clinical significance of this finding is not clear, and whether this occurs to meet the increased metabolic demands remains speculative. Irrespective of the underlying pathophysiology, our data point towards a possible relationship between metabolic derangements, such as elevated lactate or a high LP ratio, and increased risk of death in ICU COVID-19 patients. The former is related to hypoxic and non-hypoxic conditions, while the latter reflects exclusively the redox status of hypo-perfused tissues. Finally, in our sample, higher IL-6 levels were observed in patients with an abnormal metabolic pattern; this could be attributed to inflammatory mediators involved in the stimulation of lactate production, or blood flow redistribution in COVID 19 patients in whom hepatic blood flow declines together with the capacity of the liver to utilize lactate [16].

Our study has several limitations. The study population was of moderate size, but comparable to similar studies in general ICU patients. Furthermore, the number of patients with high lactate levels was small, since we enrolled consecutive, unselected COVID-19 patients. Moreover, the sample size of the non-COVID-19 control group was much smaller than the COVID-19 study group, due to the occupation of our ICU beds by COVID-19 patients. Despite these limitations, this is the first study to investigate the potential role of the LP ratio in COVID-19 critically ill patients.

## 5. Conclusions

In conclusion, elevated lactate levels or high LP ratios on admission to the ICU might be associated with poor clinical outcome. It is possible that in critically ill COVID-19 patients, hyperlactatemia may not only be due to tissue hypoperfusion. More research is necessary to elucidate the pattern and clinical meaning of LP kinetics in this new disease, and to examine the relevant pathophysiological implications.

## Figures and Tables

**Table 1:** Clinical characteristics and laboratory data in critically ill COVID-19 and non-COVID-19 patients on admission to the ICU. Patients’ outcome and demographics are also shown.

Characteristics	Critically-Ill COVID-19	Critically-Ill Non-COVID-19	*p*-Value	Effect Size
Number of patients, N	63	17		
Age (years), (mean ± SD)	63 ± 12	63 ± 12	0.97	0
Sex, N (%)			0.07	0.21
Male	48 (76.2%)	9 (52.9%)		
Female	15 (23.8%)	8 (47.1%)		
BMI (kg/m^2^), (median, IQR)	26.0 (24.5–27.8)	26.0 (24.4–30.2)	0.80	0.03
Comorbidities, N (%)	44 (69.8%)	9 (52.9%)	0.25	0.18
Diagnosis, N (%)			<0.0001 *	0.64
Medical	62 (98.4%)	8 (47.1%)		
Surgical/Trauma	1 (1.6%)	9 (52.9%)		
Characteristics on ICU admission				
APACHE II score, (median, IQR)	14 (12–17)	19 (15–20)	0.053	0.22
SOFA score, (median, IQR)	8 (7–8)	8 (7–9)	0.43	0.09
ICU admission lactate (mmol/L), (median, IQR)	1.30 (1.00–1.80)	1.10 (0.85–1.85)	0.60	0.06
Mean lactate (mmol/L), (median, IQR)	1.38 (1.13–1.68)	1.25 (0.88–1.68)	0.42	0.09
Max lactate (mmol/L), (median, IQR)	1.60 (1.30–2.00)	1.50 (1.10–2.50)	0.58	0.06
ICU admission LP ratio, (median, IQR)	4.72 (3.27–7.61)	9.18 (5.55–16.50)	0.005 *	0.36
Mean LP ratio, (median, IQR)	8.57 (5.72–11.52)	12.65 (6.31–16.25)	0.22	0.14
Max LP ratio, (median, IQR)	14.58 (10.16–23.75)	15.91 (9.85–27.62)	0.58	0.06
Mean vasopressor dose (µg/kg/min), (median, IQR)	0.16 (0.04–0.30)	0.28 (0.16–0.40)	0.03 *	0.25
Max vasopressor dose (µg/kg/min), (median, IQR)	0.25 (0.08–0.55)	0.45 (0.31–0.78)	0.01 *	0.28
Septic shock, N (%)	14 (22.2%)	5 (29.4)	0.14	0.07
Laboratory data				
pH, (mean ± SD)	7.36 ± 0.09	7.34 ± 0.06	0.3	0.24
Hemoglobin, (mean ± SD)	11.7 ± 1.7	9.9 ± 2.0	0.001 *	1.02
Hematocrit, (mean ± SD)	35.8 ± 4.7	30.1 ± 6.5	<0.0001 *	1.11
Red blood cell count (million cells per μL), (median, IQR)	4.13 (3.79–4.45)	3.47 (3.14–4.07)	0.003 *	0.35
White blood cell count (per μL), (median, IQR)	10,300 (8200–13,200)	14,910 (11,145–20,525)	0.002 *	0.34
Platelets (per μL), (median, IQR)	210,000 (164,000–270,000)	208,000 (168,500–303,000)	0.49	0.08
Creatinine (mg/dL), (median, IQR)	0.9 (0.8–1.2)	0.7 (0.5–1.1)	0.06	0.21
Total bilirubin (mg/dL), (median, IQR)	0.6 (0.4–0.8)	0.6 (0.5–0.7)	0.93	0.01
CRP (mg/dL), (median, IQR)	15.0 (8.9–21.2)	14.6 (7.8–19.2)	0.56	0.06
LDH (U/L), (median, IQR)	497 (379–702)	254 (214–463)	0.001 *	0.36
Na^+^ (mEq/L), (mean ± SD)	139.5 ± 6.2	142.7 ± 10.0	0.10	0.45
Cytokines				
IL-6 (pg/mL), (median, IQR)	16.1 (8.3–42.8)	36.6 (27.5–98.6)	0.01 *	0.28
IL-8 (pg/mL), (median, IQR)	54.7 (30.4–90.9)	61.6 (17.2–94.6)	0.85	0.02
IL-10 (pg/mL), (median, IQR)	11.4 (4.3–27.1)	11.0 (1.3–25.6)	0.50	0.19
TNF-α (pg/mL), (median, IQR)	49.1 (27.1–78.7)	32.8 (26.0–37.9)	0.12	0.18
Outcomes				
LoS in the ICU (days), (median, IQR)	24 (14–38)	19 (9–32)	0.21	0.14
ICU mortality, N (%)	26 (41.3%)	5 (29.4%)	0.42	0.1

*—p-value < 0.05. Data are expressed as number of patients (N), percentages of total related variable (%) and mean ± SD for normally distributed variables and median (IQR) for skewed data. For differences between the 2 groups, either the Student’s *t*-test for normally distributed data, the Mann-Whitney test for skewed data, or the chi-square test for qualitative variables was used. The Hedges’ g effect size was calculated for normally distributed data, Cohen’s d for skewed data, and phi for qualitative variables. The vital signs listed are the most abnormal recorded during the 24 h post-ICU admission. Laboratory data were measured once (within 24 h from ICU admission), apart from lactate and pyruvate, which were measured at four time-points. Definition of abbreviations: APACHE—Acute physiology and chronic health evaluation; CRP—C-reactive protein; ICU—Intensive care unit; IL—Interleukin; LDH—Lactate dehydrogenase; LoS—Length of stay; LP—Lactate-to-pyruvate; SOFA—Sequential organ failure assessment; TNF—Tumor necrosis factor.

**Table 2:** ICU admission clinical characteristics and laboratory data in survivors and non-survivors among critically ill COVID-19 patients.

Characteristics	Survivors	Non-Survivors	*p*-Value	Effect Size
Number of patients, N	37	26		
Age (years), (mean ± SD)	60 ± 10	68 ± 11	0.006 *	0.77
Sex, N (%)			0.24	0.17
Male	26 (70.3%)	22 (84.6%)		
Female	11 (29.7%)	4 (15.4%)		
BMI (kg/m^2^), (median, IQR)	25.7 (24.5–27.6)	26.2 (24.43–28.65)	0.38	0.11
Comorbidities, N (%)	26 (70.3%)	20 (76.9%)	0.77	0.07
APACHE II score, (median, IQR)	12 (11–16)	16 (13–21)	0.002 *	0.38
SOFA score, (median, IQR)	8 (7–8)	8 (7–9)	0.15	0.23
ICU admission lactate (mmol/L), (median, IQR)	1.20 (0.90–1.65)	1.45 (1.28–1898)	0.01 *	0.32
ICU admission LP ratio, (median, IQR)	4.04 (2.92–5.85)	5.82 (3.79–8.60)	0.056	0.24
D-dimers (µg/mL), (median, IQR)	1.09 (0.65–3.32)	1.06 (0.74–3.38)	0.71	0.05
CRP (mg/dL), (median, IQR)	14.5 (7.8–19.7)	19.3 (9.1–23.5)	0.10	0.21
LDH (U/L), (median, IQR)	513 (404–682)	460 (339–705)	0.39	0.11

*—*p*-value < 0.05. Critically ill COVID-19 patients were assigned to 2 groups based on ICU mortality. Data are expressed as number of patients (N), percentages of total related variable (%) and mean ± SD for normally distributed variables and median (IQR) for skewed data. For differences between the 2 groups, either the Student’s *t*-test for normally distributed data, the Mann-Whitney test for skewed data, or the chi-square test for qualitative variables was used. The Hedges’ g effect size was calculated for normally distributed data, Cohen’s d for skewed data, and phi for qualitative variables. Laboratory data were measured on ICU admission. Definition of abbreviations: APACHE—Acute physiology and chronic health evaluation; CRP—C-reactive protein; ICU—Intensive care unit; LDH—Lactate dehydrogenase; SOFA—Sequential organ failure assessment.

**Table 3:** Odds ratios and 95% confidence intervals for the possible prognostic factors of mortality in our cohort of critically ill COVID-19 patients.

Variables	Univariate Model			Multivariate Model		
	OR	95% CI	*p*	OR	95% CI	*p*
Age (years)	1.071	1.017–1.127	0.010 *	1.053	0.991–1.118	0.093
APACHE II score	1.190	1.048–1.352	0.007 *	1.142	0.986–1.323	0.077
ICU admission lactate (mmol/L)	3.747	1.167–12.028	0.026 *	1.968	0.420–9.217	0.390
Metabolic pattern						
Normal	Ref. value			Ref. value		
Abnormal	6.447	1.216–34.173	0.029 *	0.390	0.046–3.329	0.389

*—*p* < 0.05. A univariate logistic regression model was used to evaluate the association of age, APACHE II score, ICU admission lactate levels, and the metabolic pattern with mortality risk. Afterwards, a multivariate logistic regression model was performed to evaluate the association of the metabolic pattern with ICU mortality risk, in the presence of potential confounders, namely age, ICU admission lactate levels, and APACHE II score. A normal metabolic pattern was defined as lactate ≤ 2 mmol/L and lactate-pyruvate ratio ≤ 10. Definitions of abbreviations, APACHE—Acute physiology and chronic health evaluation; ICU—Intensive care unit.

## Data Availability

Data related to the study are available by the authors upon reasonable request.
